# The Kerbernez long-term experiment: A dataset on crop yield and soil organic matter evolution in forage crop rotations and permanent grasslands in a temperate oceanic climate

**DOI:** 10.1016/j.dib.2024.111259

**Published:** 2024-12-25

**Authors:** Anne-Isabelle Graux, Alice Cadéro, Fabien Ferchaud, Françoise Vertès

**Affiliations:** a PEGASE, INRAE, Institut Agro, 16 Le Clos, Saint-Gilles 35590, France; b BioEcoAgro Joint Research Unit, INRAE, Université de Liège, Université de Lille, Université de Picardie Jules Verne, 02000 Barenton-Bugny, France; c UMR Eco&Sols, Université de Montpellier, CIRAD, INRAE, IRD, Institut Agro Montpellier, Montpellier, France; d UMR SAS, INRAE, Institut Agro, 35 000 Rennes, France

**Keywords:** France, Brittany, Forage crop rotations, Grassland, Soil organic carbon and nitrogen, Modelling

## Abstract

Forage crop rotations including grasslands, common in dairy systems, are known to ensure good productivity and limit the decrease of soil organic matter frequently observed in permanent arable land. A dataset was built to compile data from the Kerbernez long-term experiment, conducted in Brittany(France) from 1978 to 2005. This experiment compared the effect of different forage crop rotations fertilized with ammonium nitrate and/or slurry, with or without grassland, on forage production (quantity, quality) and changes in soil physio-chemical characteristics. These forage crop rotations were based on silage maize and cut monospecific grasslands of Italian ryegrass (*Lolium multiflorum* L.) or perennial ryegrass (*Lolium perenne* L.). More precisely, the experiment compared silage maize monocultures, rotations with silage maize and Italian ryegrass established for 6 to 18 months, and rotations with silage maize and perennial ryegrass established for three to more than 10 years. They are representative of the forage crop rotations and permanent grasslands that were at the heart of Brittany's forage revolution in the 1970s. The dataset includes information about the climate and soil conditions, the management of crops and grasslands, the evolution of topsoil organic carbon and nitrogen stocks, the inter-annual variations in crop and grassland dry matter yields and nitrogen contents. The dataset also includes characterisation of soil structural stability, particle-size soil organic matter fractions and potential soil carbon and nitrogen mineralisation at the end of the trial. It consists of fourteen csv files. This dataset can be used for a variety of purposes, namely for assessing the ability of mechanistic models to simulate soil organic matter dynamics and associated fluxes, and to estimate the influence of grassland presence and duration in forage crop rotations on such fluxes.

Specifications Table

**Table:** 

Subject	Agronomy and Crop Science.
Specific subject area	Crop and grassland yields, soil organic carbon and nitrogen in relation to the presence and duration of grassland in the rotation and fertilisation practices
Type of data	Tables, Figures, R code
Data collection	The raw and calculated data were collected on a long-term experimental set-up located in France (Brittany) directly from the INRAE researchers responsible for or having worked on this experimental set-up. Data useful for a modelling exercise with the STICS soil-crop model were checked, cleaned and pooled in a dataset composed of fourteen csv files. Each file presents one type of information: daily weather, management of forage crops, forage crop dry matter yield and N content, soil initial conditions, topsoil organic C and total N stocks, final soil conditions including soil bulk density, potential soil C and N mineralisation, soil organic matter fractions and stability of soil aggregates.
Data source location	ORE AgrHyS Observatory, INRAE, Institut Agro, Kerbernez site (47°56′49.2″ N 4°07′33.6″ W, 36 m a.s.l.), 29,700 Plomelin, Brittany, France*.*
Data accessibility	Repository name: Recherche Data Gouv Data identification number: DOI dataset: 10.57745/P8NNZK Direct URL to data: https://doi.org/10.57745/P8NNZK
Related research article	H. Clivot, J.C. Mouny, A. Duparque, J.L. Dinh, P. Denoroy, S. Houot, F. Vertès, R. Trochard, A. Bouthier, S. Sagot, B. Mary. Modeling soil organic carbon evolution in long-term arable experiments with AMG model. Environ. Model. Softw., 118 (2019), 99–113. https://doi.org/10.1016/j.envsoft.2019.04.004

## 1 Value of the Data

• Monitoring the soil organic matter dynamics and improving knowledge about its main drivers remain a challenge. This dataset provides data on crop and grassland yields and dynamics of soil organic carbon and total nitrogen stocks for different types of land use, along with information on crop and grassland management, initial soil characteristics, climate conditions and plant health. It was built from a long-term experiment (27 years) with a randomized complete block design. Additional measurements were made at the end of the trial to characterize soil aggregate stability, particle-size soil organic matter fractions and potential soil C and N mineralization. This type of data is rare.
• This dataset is of interest to agronomists, soil scientists and modellers.
• It can be used for a variety of applications, for instance, i) within a meta-analysis about soil organic matter dynamics; ii) for assessing the ability of crop-soil or soil models (e.g. AMG, STICS, PaSim) to simulate soil organic matter dynamics and associated fluxes, and to be confident about the results they deliver when used to test scenarios; iii) to estimate the influence of grassland presence and duration in forage crop rotations on soil organic matter dynamics and associated fluxes.

## 2 Background

After the Second World War, Brittany underwent a major development in agriculture, known as the “green revolution”, based on increasing the individual milk production of cows, and made possible by access to soil liming, cheap chemical fertilisers and by the massive use of pure grass in grazing systems or permanent grasslands. In the 1970s, maize species adapted to temperate oceanic climate were rapidly integrated into forage systems. Development of cows/pigs/poultry breeding led to large slurry availability, and even local excess. The Kerbernez long-term experiment, initiated in 1978, aimed to compare the short- and long-term effects of 11 forage rotations (some of which had a variant depending on the rotation head at the start of the experiment) present in these forage systems, on crop and soil characteristics, using a set of regular observations**.** This data paper focuses on the seven rotations including only maize and/or grass and excludes the four rotations with other crops (wheat or grain legumes). In 1991, only part of the experiment was extended until 2005 to quantify long-term changes in the soil. This dataset was used to characterize and simulate changes in soil organic carbon stocks between 1970 and 2015 using the AMG model [1]. More recently, it has been used to assess the ability of the STICS model to simulate changes in organic carbon and nitrogen stocks in permanent and temporary grassland soils. These modelling exercises provided an opportunity to collect and consolidate the data from this experiment [2].

## 3 Data Description

**Fig. 1:**
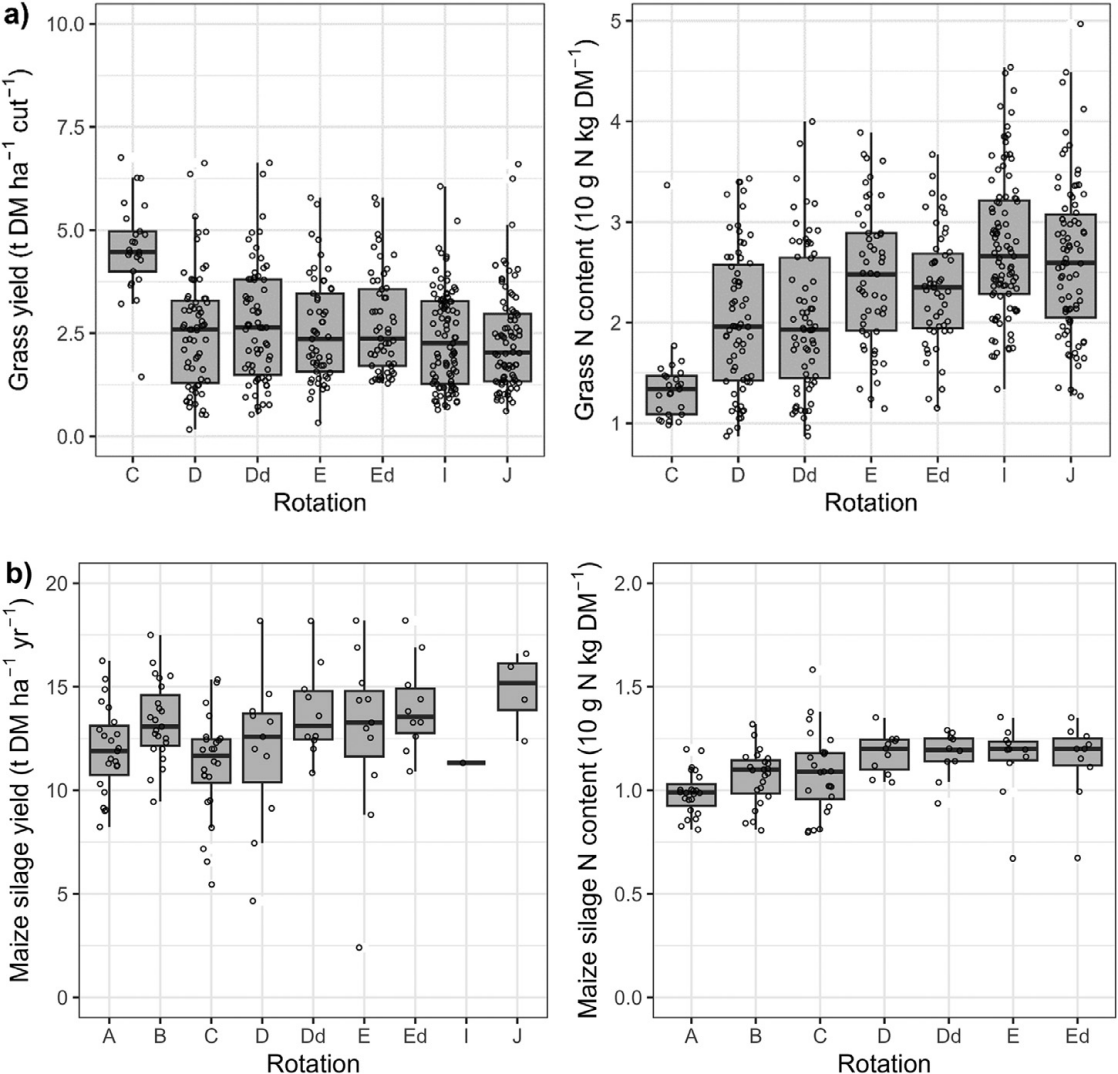
Distribution of average dry matter yields and N content of a) grassland and b) silage maize by rotation during the 27 years of the Kerbernez experiment. The values are available for each cut in the case of grassland. The dots indicate the different values measured during the experiment.

**Fig. 2:**
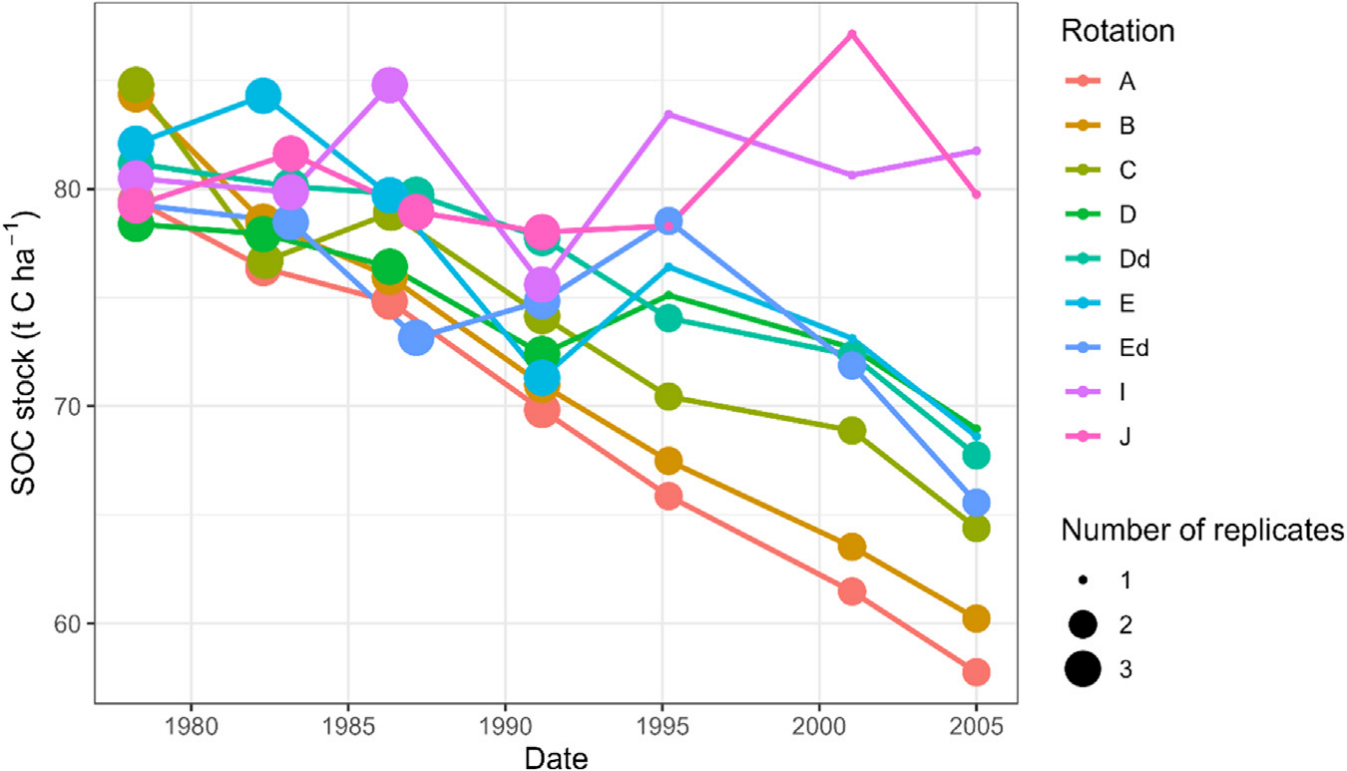
Dynamics of soil organic carbon (SOC) stocks in 0–25 cm for the different rotations of the long-term Kerbernez experiment (1978–2005). The size of dots is proportional to the number of replicates.

**Fig. 3:**
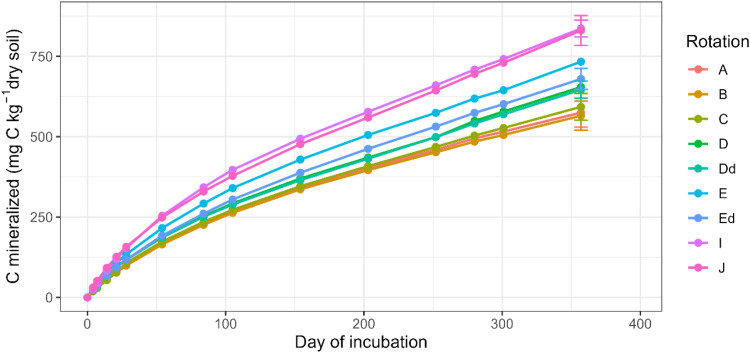
Cumulated carbon mineralised in incubated soils (0–25 cm) at the end (2005) of the Kerbernez long-term experiment according to the rotation. Error bars correspond to the standard error of Cumulated carbon mineralised at the end of incubation.

**Fig. 4:**
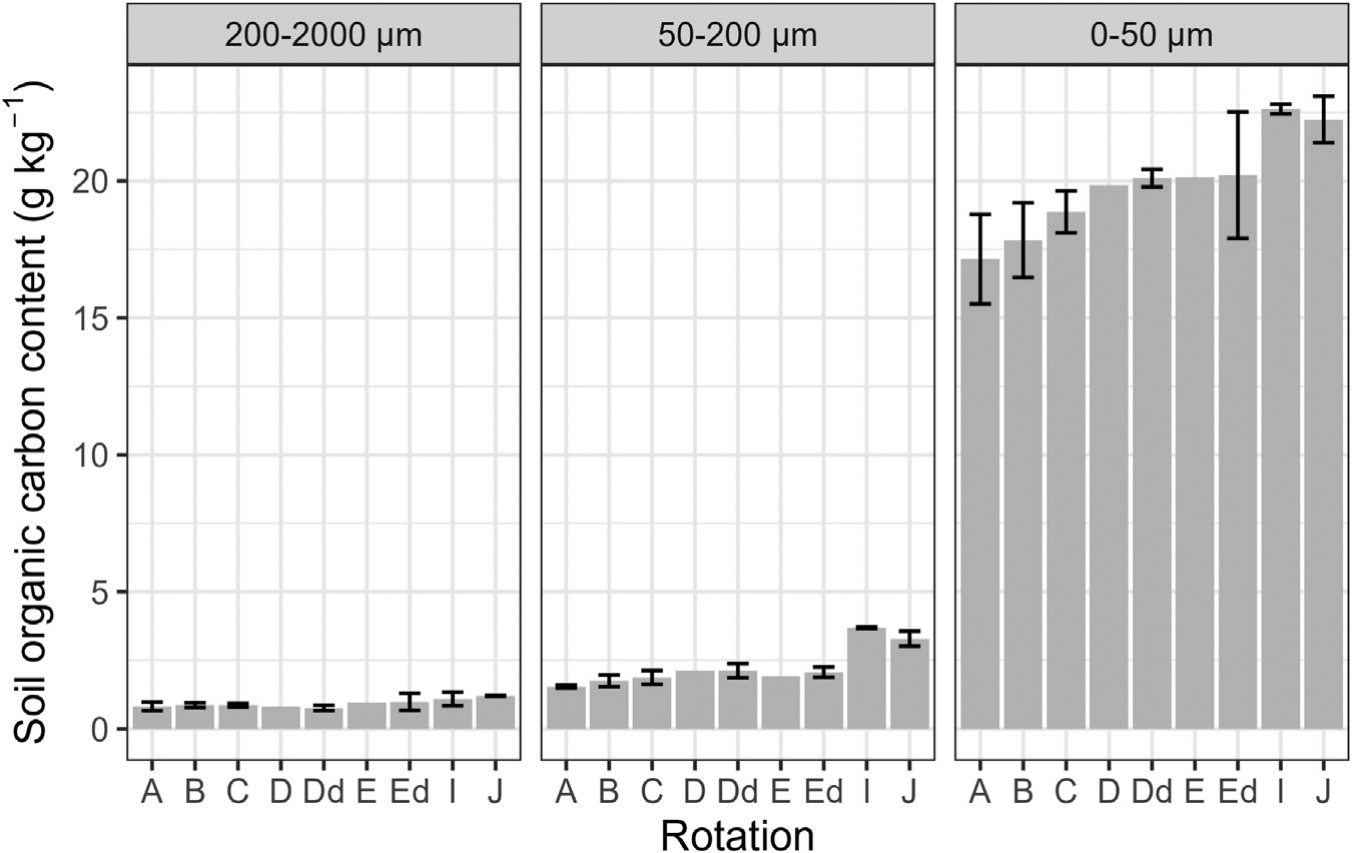
Repartition of the topsoil organic carbon content into three pools, i.e. the coarse particulate organic matter (POM) fraction (200–2000 µm), the fine POM fraction (50–200 µm) and the mineral-associated organic matter (MAOM) fraction (0–50 µm), at the end (2005) of the Kerbernez long-term experiment according to the rotation.

**Fig. 5:**
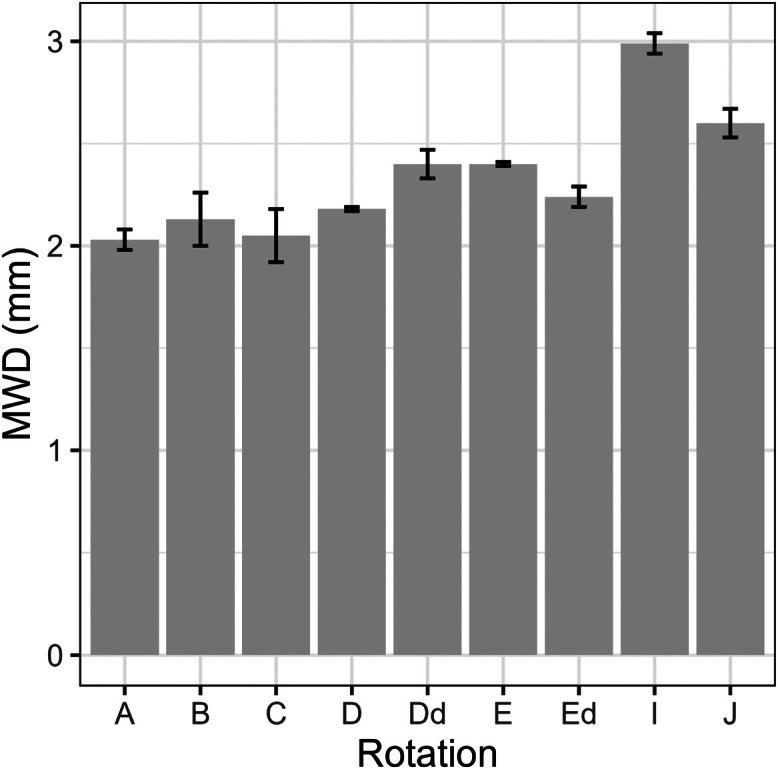
Soil aggregate mean-weight diameter (MWD) estimated at the end (2005) of the Kerbernez long-term experiment according to the rotation.

**Fig. 6:**
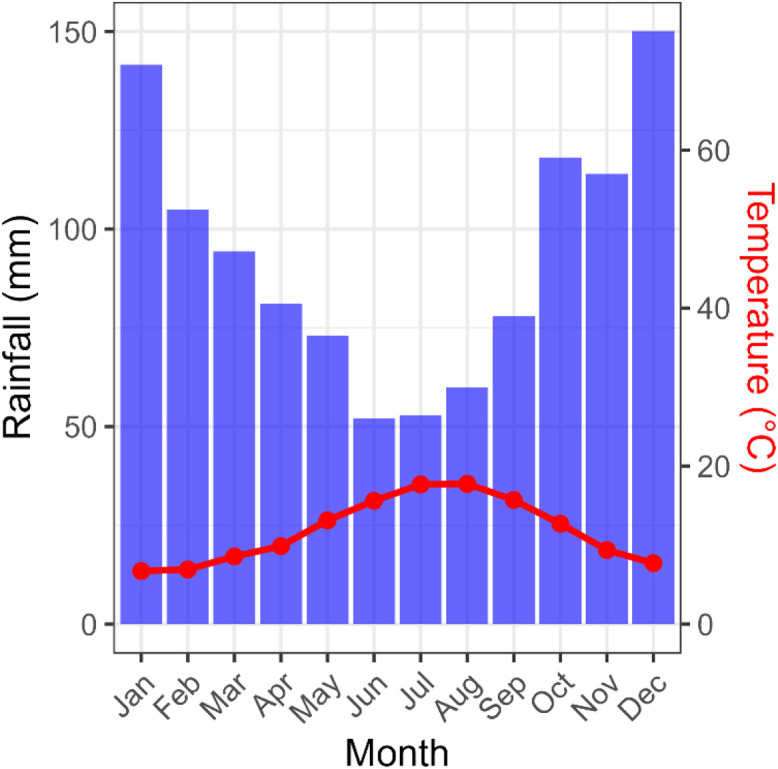
Ombrothermic diagram indicating the monthly average temperature (in red) and rainfall (in blue) pattern at the Kerbernez site. The weather variables are averaged over the 1978–2005 period.

**Fig. 7:**
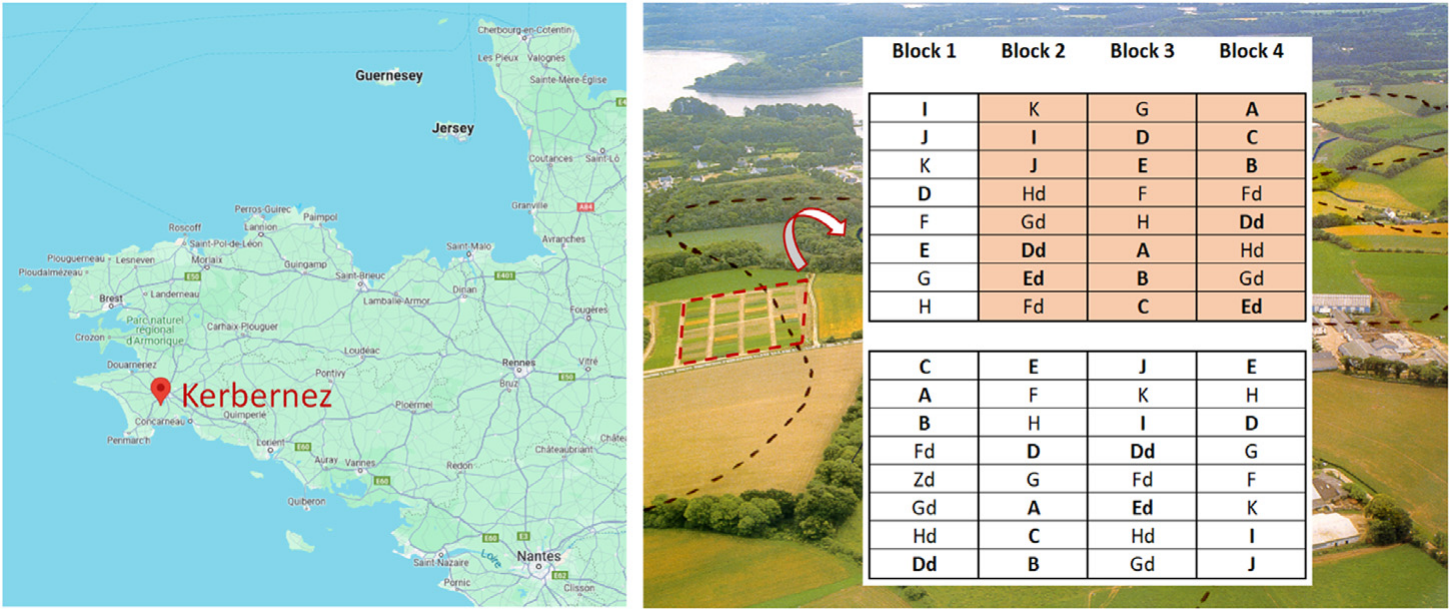
Location and experimental design of the Kerbernez trial. The data from block 1 has not been taken into account due to an after-effect of the presence of a hedge. Only the orange part of the experiment was kept after 1991. The names of rotations in bold are those that appear in this dataset.

**Fig. 8:**
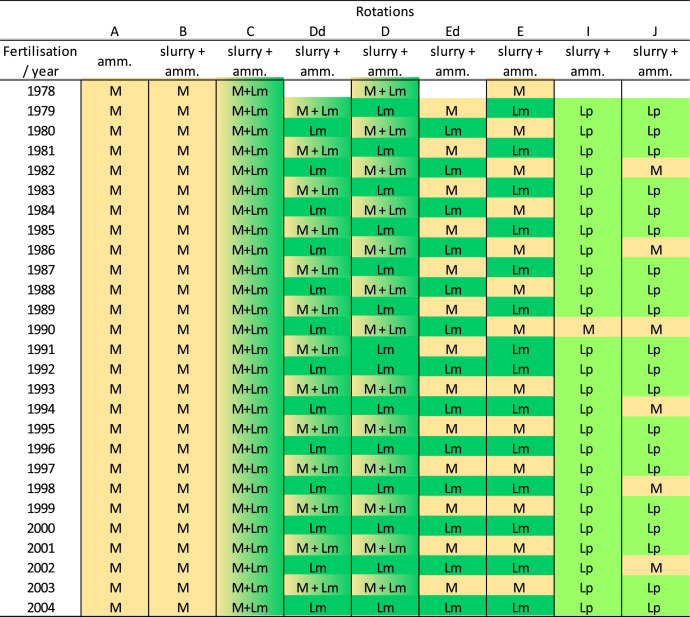
Description of the forage crop rotations and permanent grasslands in the Kerbernez long-term experiment. amm. = ammonium nitrate; M = Maize; Lm = *Lolium multiflorum* L.; Lp = *Lolium perenne* L.

This article includes tables and figures that describe data of the Kerbernez long-term experiment, including, during the experiment, the distribution of forage crop dry matter yields and N contents (Fig. 1) and the dynamics of soil organic C stocks in the different forage crop rotations (Fig. 2); and at the end of the experiment, the potential soil C and N mineralisation (Fig. 3), the particle-size soil organic matter fractions (Fig. 4) and the soil structural stability (Fig. 5). It also includes a description of the average intra-annual temperature and rainfall pattern (Fig. 6), the location and experimental design of the experiment (Fig. 7), and a description of the forage crop rotations and permanent grasslands included in the experiment (Fig. 8), and finally a summary of observations made at the start, during and at the end of the experiment (Table 1).

**Table 1:** Summary of observations made at the start, during and at the end of the experiment and layers concerned for observations made on the soil.

Measurement	Start of the experiment (1978)	During the experiment	End of the experiment (2005)
Soil texture	0–25 cm	–	–
Soil pH	0–25 cm	–	–
Soil bulk density	–	–	0–25 cm
Soil structural stability	–	–	0–25 cm
Soil C and N concentration	0–25 cm	0–25 cm	0–25 cm
Crop and grassland dry matter yield	–	At each harvest or cut event	–
Crop and grassland N content	–	At each harvest or cut event	–
Crop and grassland health (weeds, pests and diseases)	–	1 to 4 observations according to years	–
Total N, ammoniacal N and dry matter content of slurry	–	Before each slurry application	–

The Kerbernez dataset consists of fourteen csv files that contain raw and computed data (Table 2). It includes data on daily weather, management of forage crop, forage crop dry matter yield and N content, soil initial conditions, topsoil organic C and total N stocks, final soil conditions including soil bulk density, potential soil C and N mineralisation, soil organic matter fractions, and stability of soil aggregates. The dataset is available via the French DataGouv repository. Additional data in French and in PDF format on encoding observations and on crop health monitoring are also stored in this repository but are not described in this paper because they are not computerised or in English.

**Table 2:** Description of the contents of each of the files included in the Kerbernez dataset.

File name	Variable name	Content
Kerbernez_climate.csv	Date	Date
	Tavg	Daily average temperature (°C)
	tmin	Daily minimum temperature (°C)
	tmax	Daily maximum temperature (°C)
	srad	Daily global radiation (mJ m^-2^)
	ETP	Daily Penman evapotranspiration (mm)
	rain	Daily rainfall (mm)
Kerbernez_management_crop_sowing.csv	rotation	Rotation identifier
	year	Year
	crop	Name of the forage crop
	date	Date of crop sowing
	sowing_depth	Sowing depth (cm)
	sowing_density	Sowing density (plant m^-2^)
Kerbernez_management_grassland_ploughing_up.csv	rotation	Rotation identifier
	year	year
	crop	Name of the forage crop
	date	Date of grassland ploughing up
Kerbernez_management_mineral_N_fertilisation.csv	rotation	Rotation identifier
	year	year
	crop	Name of the forage crop
	date	Date of mineral nitrogen fertiliser application
	mineral_N_fert_type	Type of mineral nitrogen fertiliser
	mineral_N_amount	Amount of applied mineral nitrogen (kg N ha^-1^)
Kerbernez_management_slurry_application.csv	rotation	Rotation identifier
	year	year
	crop	Name of the forage crop
	date	Date of slurry application
	slurry_type	Type of slurry
	slurry_amount	Amount of applied fresh slurry (t FW ha^-1^)
	slurry_C_content	Slurry C content (% DW = 10 g C kg^-1^ DW)
	slurry_Norg_content	Slurry Norg content (% DW = 10 g Norg kg^-1^ DW)
	slurry_Nmin_content	Slurry mineral N content (% FW = 10 g Nmin kg^-1^ FW)
	slurry_H2O_content	Slurry water content (% FW = 10 g H_2_O kg^-1^ FW)
Kerbernez_management_soil_tillage.csv	rotation	Rotation identifier
	year	Year
	crop	name of the forage crop
	date	date of soil tillage
	type_of_tillage	type of soil tillage
	tillage_depth	tillage depth (cm)
Kerbernez_plant_grassland_yield.csv	rotation	rotation identifier
	year	year
	crop	name of the forage crop
	date	date of grassland cut
	avg_DM_yield	average grassland dry matter yield (t DM ha^-1^ cut^-1^)
	sd_DM_yield	standard deviation of grassland dry matter yield (t DM ha^-1^ cut^-1^)
	n_rep_DM_yield	number of replicates used to compute avg_DM_yield and sd_DM_yield
	avg_crop_N_content	average N content in grassland dry matter (% DM = 10 g N kg^-1^ DM)
	sd_crop_N_content	standard deviation of N content in grassland dry matter (% DM)
	n_rep_crop_N_content	number of replicates used to compute avg_crop_N_content and sd_crop_N_content
Kerbernez_plant_maize_yield.csv	rotation	rotation identifier
	year	year
	crop	name of the forage crop
	date	date of crop harvest
	avg_DM_yield	average silage maize dry matter yield (t DM ha^-1^ yr^-1^)
	sd_DM_yield	standard deviation of silage maize dry matter yield (t DM ha^-1^ yr^-1^)
	n_rep_DM_yield	number of replicates used to compute avg_DM_yield and sd_DM_yield
	avg_crop_N_content	average N content in silage maize dry matter (% DM = 10 g N kg^-1^ DM)
	sd_crop_N_content	standard deviation of N content in silage maize dry matter (% DM)
	n_rep_crop_N_content	number of replicates used to compute avg_crop_N_content and sd_crop_N_content
Kerbernez_soil_bulk_density	rotation	rotation identifier
	year	year
	crop	soil layer (lower limit-upper limit in cm)
	date	date of soil sampling
	n_rep_soil_density	number of replicates used to compute average and standard deviation values (columns prefixed with “avg” and “sd”)
	avg_BD_global	average global soil bulk density (fine soil + pebbles mass divided by total soil volume) (g cm^-3^)
	sd_BD_global	standard deviation of global soil bulk density (g cm^-3^)
	avg_BD_pebbles	average bulk density of pebbles (mass of pebbles divided by volume of pebbles) (g cm^-3^)
	sd_BD_pebbles	standard deviation of bulk density of pebbles (g cm^-3^)
	avg_BD_fine_soil	average fine soil bulk density (mass of fine soil divided by total soil volume) (g cm^-3^)
	sd_BD_fine_soil	standard deviation of fine soil bulk density (g cm^-3^)
	avg_pebbles_mass_fract	average mass fraction of pebbles (> 2 mm) in the soil (%)
	sd_pebbles_mass_fract	standard deviation of mass fraction of pebbles (> 2 mm) in the soil (%)
	avg_pebbles_vol_frat	average volume fraction of pebbles (> 2 mm) in the soil (%)
	sd_pebbles_vol_fract	standard deviation of volume fraction of pebbles (> 2 mm) in the soil (%)
	avg_fine_soil_mass_fract	average mass fraction of fine soil (<2 mm) in the soil (%)
	sd_fine_soil_mass_fract	standard deviation of mass fraction of fine soil (<2 mm) in the soil (%)
	avg_fine_soil_vol_fract	average volume fraction of fine soil (<2 mm) in the soil (%)
	sd_fine_soil_vol_fract	standard deviation of volume fraction of fine soil (<2 mm) in the soil (%)
Kerbernez_soil_CN_dynamics.csv	rotation	rotation identifier
	year	year
	crop	name of the forage crop or bare soil period
	layer	soil layer (lower limit-upper limit in cm)
	date	date of soil measurement
	avg_SOC	average soil organic carbon content (g C kg^-1^)
	sd_SOC	standard deviation of soil organic carbon content (g C kg-^1^)
	avg_SOC_stock	average soil organic carbon stock (t C ha^-1^)
	sd_SOC_stock	standard deviation of soil organic carbon stock (t C ha^-1^)
	n_rep_SOC_stock	number of replicates used to compute avg_SOC_stock and sd_SOC_stock
	avg_STN	average soil total nitrogen content (g N kg^-1^)
	sd_STN	standard deviation of soil total nitrogen content (g N kg^-1^)
	avg_STN_stock	average soil total nitrogen stock (t N ha^-1^)
	sd_STN_stock	standard deviation of soil total nitrogen stock (t N ha^-1^)
	n_rep_STN_stock	number of replicates used to compute avg_STN_stock and sd_STN_stock
Kerbernez_soil_CN_mineralisation.csv	rotation	rotation identifier
	year	year
	layer	soil layer (lower bound-upper bound in cm)
	day_incubation	number of the day of incubation
	normalized_day	normalized day (at 15 °C and 100 % field capacity)
	n_rep_CN_mineralisation	number of replicates used to compute average and standard deviation values (columns prefixed with “avg” and “sd”)
	avg_SMN	average soil mineral nitrogen content (mg N kg^-1^ dry soil)
	sd_SMN	standard deviation of soil mineral nitrogen content (mg N kg^-1^ dry soil)
	avg_mineralized_N	average soil nitrogen mineralisation over the period (mg N kg^-1^ dry soil)
	sd_mineralized_N	standard deviation of soil nitrogen mineralisation over the period (mg N kg^-1^ dry soil)
	avg_cum_mineralized_N	average cumulated soil nitrogen mineralisation (mg N kg^-1^ dry soil)
	sd_cum_mineralized_N	standard deviation of cumulated soil nitrogen mineralisation (mg N kg^-1^ dry soil)
	avg_mineralized_C	average soil carbon mineralisation over the period (mg C kg^-1^ dry soil)
	sd_mineralized_C	standard deviation of soil carbon mineralisation over the period (mg C kg^-1^ dry soil)
	avg_cum_mineralized_C	average cumulated soil carbon mineralisation (mg C kg^-1^ dry soil)
	sd_cum_mineralized_C	standard deviation of cumulated soil carbon mineralisation (mg C kg^-1^ dry soil)
Kerbernez_soil_init_conditions	rotation	rotation identifier
	year	year
	bedrock_depth	soil bedrock depth (cm)
	layer	soil layer (lower bound-upper bound in cm)
	clay	clay content (%)
	silt	silt content (%)
	sand	sand content (%)
	fine_silt	fine silt content (%)
	coarse_silt	coarse silt content (%)
	fine_sand	fine sand content (%)
	coarse_sand	coarse sand content (%)
	water_pH	pH measured in water (-)
Kerbernez_soil_OM_fractions.csv	rotation	rotation identifier
	Year	year
	Layer	soil layer (lower bound-upper bound in cm)
	n_rep_fractions	number of replicates used to compute average and standard deviation values (columns prefixed with “avg” and “sd”)
	avg_C_200-2000	average soil organic carbon content in the 200–2000 µm fraction (g kg^-1^)
	sd_C_200-2000	standard deviation of soil organic carbon content in the 200–2000 µm fraction (g kg^-1^)
	avg_C_50-200	average soil organic carbon content in the 50–200 µm fraction (g kg^-1^)
	sd_C_50–200	standard deviation of soil organic carbon content in the 50–200 µm fraction (g kg^-1^)
	avg_C_0–50	average soil organic carbon content in the 0–50 µm fraction (g kg^-1^)
	sd_C_0-50	standard deviation of soil organic carbon content in the 0–50 µm fraction (g kg^-1^)
	avg_N_200-2000	average soil nitrogen content in the 200–2000 µm fraction (g kg^-1^)
	sd_N_200-2000	standard deviation of soil nitrogen content in the 200–2000 µm fraction (g kg^-1^)
	avg_N_50-200	average soil nitrogen content in the 50–200 µm fraction (g kg^-1^)
	sd_N_50-200	standard deviation of soil nitrogen content in the 50–200 µm fraction (g kg^-1^)
	avg_N_0-50	average soil nitrogen content in the 0–50 µm fraction (g kg^-1^)
	sd_N_0-50	standard deviation of soil nitrogen content in the 0–50 µm fraction (g kg^-1^)
Kerbernez_soil_structural_stability.csv	Rotation	rotation identifier
	Year	year
	Layer	soil layer (lower bound-upper bound in cm)
	n_rep_soil_structural_stability	number of replicates used to compute average and standard deviation values (columns prefixed with “avg” and “sd”)
	avg_MWD_FW	average soil aggregate mean-weight diameter after fast wetting (mm)
	sd_MWD_FW	standard deviation of soil aggregate mean-weight diameter after fast wetting (mm)
	avg_MWD_SW	average soil aggregate mean-weight diameter after slow wetting (mm)
	sd_MWD_SW	standard deviation of soil aggregate mean-weight diameter after slow wetting (mm)
	avg_MWD_MB	average soil aggregate mean-weight diameter after mechanical breakdown (mm)
	sd_MWD_MB	standard deviation of soil aggregate mean-weight diameter after mechanical breakdown (mm)
	avg_MWD	average soil aggregate mean-weight diameter according to the three tests (mm)
	sd_MWD	standard deviation of soil aggregate mean-weight diameter according to the three tests (mm)

## 4 Experimental Design, Materials and Methods

### 4.1 Material and methods of the experiment

#### 4.1.1 Location and description of the experimental design

This long-term experiment [[3], [4], [5], [6]] started in 1978 and was located in Kerbernez, in the French Brittany region (47°56′49.2″ N 4°07′33.6″ W, 36 m a.s.l.). The climate was temperate oceanic. The mean annual temperature was 11.8 °C and annual precipitation was about 1120 mm (Fig. 6).

The soil had a water holding capacity of about 190 mm and a sandy loam texture (on average 16 % clay, 39 % silt and 45 % sand) [7]. It was moderately acidic (pH=5.5) and relatively rich in organic matter (4.6 % on average in 0–25 cm) with slight initial differences between the rotations (initial SOC stock of 81.0 ± 2.3 t C ha^-1^ in 0–25 cm).

The experiment aimed to study the effect of the nature of the forage crop rotation (presence and duration of grasslands in the rotation) and of fertilisation on the evolution of forage crop yields and soil organic matter. It was a randomized complete block design, with initially 4 blocks. Each block measured 32 × 144 m and was bisected in the middle by an alleyway. Each block was divided into 32 × 9 m plots, each corresponding to a repetition of a forage crop rotation. Rotations were followed for 27 years (1978–2005). Each of these rotations was repeated 3 to 4 times until 1991, then only 1 to 2 times from 1991 onwards. Indeed, a part of the experiment was abandoned in 1991 following the retirement of the person in charge of the experimental trial and due to insufficient labour time available to monitor the entire experimental trial as originally designed (Fig. 7).

Eleven forage crop rotations were studied in this trial, graded from A to K. In this dataset, we have only compiled data for seven of these rotations (two of them with a variant depending on the rotation head at the start of the experiment and suffixed with “d”). Crop forage rotations include: i) 2 silage maize monocultures, rotation A receiving only ammonium nitrate, and B receiving both ammonium nitrate and cattle or pig slurry; ii) 5 rotations of silage maize and Italian ryegrass (*Lolium multiflorum* L.) established for 6 to 18 months (rotations C, D, Dd, E and Ed, the effect of the head of the rotation being taken into account between rotations D and Dd, and between rotations E and Ed), all receiving both ammonium nitrate and cattle or pig slurry, iv) 2 rotations of silage maize with perennial ryegrass (*Lolium perenne* L.), established permanently (> 10 years, rotation I) or temporary (3 years, rotation J) and receiving both ammonium nitrate and cattle or pig slurry.

#### 4.1.2 Crop management

The experiment was conducted using standard agricultural machinery. Rotations were carried out rather intensively with high levels of N inputs supplied in mineral and/or organic form: on average, 60 kg mineral N ha^−1^ yr^−1^ on maize, with the exception of rotation A (120 kg mineral N ha^−1^ yr^−1^), around 140 kg mineral N ha^−1^ yr^−1^ on Italian ryegrass and around 200 kg mineral N ha^−1^ yr^-1^ on perennial ryegrass; on average, 60 t of fresh slurry ha^−1^ yr^−1^ on maize (with the exception of rotation A which received only mineral fertiliser), Italian ryegrass and perennial ryegrass. Maintenance fertilisation with phosphorus and potassium was carried out on rotation A, to ensure soil fertility, because it was the only one without slurry application. In this trial, the grasslands were frequently mown with on average five cuts per year, except for rotation C for which only one mowing of the Italian ryegrass was carried out in the spring. After ploughing over 25 cm, the silage maize was sown in April with early cultivars (LG11 from 1978 to 1995, DEA from 1996 to 2001 then Anjou258) and harvested in September or October.

#### 4.1.3 Plant, soil and slurry measurements and analysis

Data were collected to characterize the inter-annual variability and intra-annual distribution of crop yields, the nitrogen content of harvested fodder, crop health, the chemical composition of the slurry spread and the physicochemical properties of soils. Various observations were made whether the experiment was at the beginning, during or at the end (see Table 1). In particular, the topsoil (0–25 cm) layer was sampled 7 times over the 27 years (including the start of the experiment) and analysed to quantify soil organic C and total N concentrations. The soil bulk density and pebble (> 2 mm) content were only estimated at the end of the trial.

Maize and grassland dry matter yields were estimated until 2002 from a sample corresponding to a strip harvested in the centre of each plot. This strip was respectively of 22.5 m^2^ and 10 m^2^ for maize and grassland. Fresh maize and grass samples were taken respectively at a cutting height of 10 and 5 cm using a motor mower. Whatever the forage crop, the harvested sample was weighed fresh in the field and a sub-sample was taken and dried at 60 °C during 48 h to determine the dry matter content and carry out mineral analyses. We only recorded the nitrogen content of the forages in the present dataset, but full mineral analyses (P, K, Ca, Na, Mg, Cu, Zn, Mn, ash content, cellulose, etc.), were carried out for all samples. In particular, dried samples were ground and analysed for total N content using the Kjeldahl method [9]. Observations, whether of yield components or plant health aspects, were carried out on side strips on either side of the central strip.

The physico-chemical properties of the soils were determined for each plot of the experiment. The soil samples were obtained by mixing eight sub-samples from each soil layer of each plot. The soil samples were then dried at 40 °C and sieved to 2 mm for physical or chemical analysis.

Soil total N was determined by the Kjeldahl [9] and/or the Dumas dry combustion method [8] methods. Total soil carbon was determined by the Dumas method or by multiplying total soil nitrogen determined by the Kjeldahl method by an average soil C/N ratio for the rotation under consideration. Soil pH was obtained in water with the method described in [10]. Soil texture was measured using five particle size fractions: clay (< 2 µm), fine silt (2–20 µm), coarse silt (20–50 µm), fine sand (50–200 µm) and coarse sand (200–2000 µm) [11].

Soil bulk density was measured once, at the end of the experiment (2005), for each experimental plot and four layers (0–10 cm, 10–25 cm, 25–35 cm and 35–50 cm), using an 8 cm diameter root auger, which cored undisturbed samples of known volume. All samples were oven dried at 105 °C, weighed, and sieved at 2 mm. Samples were then weighed again to determine the fin earth and pebble content.

At the end of the experiment in 2005, additional soil samples (0–25 cm) were taken and sieved at 2 mm to remove fragments of plant residues and pebbles. Soil samples from rotations D and Dd on the one hand and E and Ed on the other hand were mixed, as they corresponded to the same rotation but started with a different rotation head. They were incubated in the laboratory at 15 °C and at constant soil water content (90 % of field capacity) to determine the potential C and N mineralization for 396 days. Mineralised C was continuously monitored by CO_2_ trapping. Mineralised N was determined at regular intervals on soil samples by extracting mineral N with KCl solution 1 N (50 ml for 20 g wet soil, agitation). Mineralisation was expressed in normalized days (i.e. days at 15 °C and 100 % field capacity) as defined by [12].

In parallel, particle size fractionation of organic matter was achieved on the same soil samples according to the physical fractionation method proposed by [13]. This fractionation process resulted in three pools: the mineral-associated organic matter (MAOM) fraction (< 50 µm), the fine particulate organic matter (POM) fraction (50–200 µm) and the coarse POM fraction (200–2000 µm). Each fraction was dried, weighted and ground. The two POM fractions were analysed for total C and N content (Dumas method), data for the finest fractions being calculated by difference with the bulk soil.

Aggregate stability was determined according to the method described by [14] and more recently standardized [15]. This method combines three disruptive tests that correspond to various wetting conditions and energies: fast wetting, slow wetting and mechanical breakdown by shaking after pre-wetting. The fragmented samples are then sieved and treated to calculate the mass proportion of each size fraction of the stable aggregates. Results were expressed as the mean-weight diameter (MWD) corresponding to the sum of the mass fraction remaining on each sieve multiplied by the mean of the inter-sieve sizes. In the table MWD expressed in mm are the mean value of the three tests.

The slurry applied on forage crops was sampled and weighed before each application. The dry matter content of slurry was measured by drying a subsample at a temperature of 103 ± 2 °C [16]. The organic matter content of slurry was determined by calcination at 450 ± 25 °C [17]. Total N and ammonium N of slurry were analyzed in raw subsamples to avoid gaseous N losses during drying. Total N was measured by the Kjeldahl method [9] and ammonium N was measured by steam distillation, using a method similar to the Kjeldahl method that volatilizes ammonium by adding a strong base to the distillation mash [18]. Ammonia volatilized during distillation is trapped in a known quantity of boric acid, and the ammonium content is measured by titration with a 0.1 N HCl solution.

### 4.2 Material and methods for building the dataset

#### 4.2.1 Data recovery, screening and gap-filling

The data relating to the protocols, experimental conditions and measurements were collected from the paper documents used to monitor the experiment and the publications of the INRAE researchers in charge of the experiment or who carried out additional analyses of the soil samples afterwards. This work of compiling and computerising the data was carried out in particular with the aim of being able to use this dataset to assess the capacity of the STICS soil-crop model to simulate soil organic matter dynamics in rotations including temporary grasslands and in permanent grasslands. Consequently, only the forage rotation data that the model was capable to simulate were retained.

Raw climate data comes from the INRAE CLIMATIK platform [19]. Climatic data from the INRAE station at Plomelin, located on the experimental site, were used to describe the site's climate. However, in case of missing data, we replaced the missing data with data from nearby INRAE climate stations. We used the data available for the station closest to the site: in this order of preference, the stations in Pluguffan (4 km from the experimental site), Quimper (9 km), Plougoulm (59 km), Guipavas (60 km), Ploudaniel (64 km) and Saint Pol de Léon (79 km). However, we respected the limiting distances given per climate variable by [20] to have a total weight of 1, namely no more than 6 km for rainfall, no more than 20 km for potential evapotranspiration and relative humidity, no more than 30 km for wind speed, no more than 50 km for minimum and maximum temperatures and finally no more than 100 km for global radiation. For the whole experimental period, 98 % of the rainfall and temperature data, 87 % of the potential evapotranspiration and global radiation data, but only 48 % of the air humidity data come from the meteorological station of the experiment (i.e. Plomelin). If required, the annual atmospheric CO_2_ concentration is available on this Météo-France repository (https://meteo.data.gouv.fr/datasets/6569b27598256cc583c917a7).

In some years (1986, 2001 and 2003), part of the information regarding the characterisation of the chemical composition of the slurry was missing. In these cases, the average value of the pig slurry or cattle slurry from the trial was used to fill in the Kerbernez_slurry_application.csv file.

#### 4.2.2 Data calculation

The data from block 1 were not taken into account in the calculations of the mean values, standard deviations and number of repetitions of the soil and plant observations because the soil conditions were found to be different from those in the other blocks due to an after-effect of the presence of a hedge. These calculations also took into account the data available, which was more numerous before 1991 than after, due to the reduction in the size of the experimental trial.

##### 4.2.2.1 Calculation of crop and grassland yields

Forage crop yields in tonnes of dry matter (DM) per hectare were estimated from the fresh biomass of the sample taken from the central strip of each of the plots, multiplied by the DM content of the sample obtained from the analyses and related to the area of the sample strip, which was itself measured.

##### 4.2.2.2 Calculation of topsoil organic C and total N stocks

We first calculated the soil total N stock (STN_stock, expressed in t N ha^-1^) in the 0–25 cm topsoil as followed:

$$STN\_stock=\frac{STN\times LT\times BD}{10}$$

withSTN, the topsoil layer N concentration (g N kg^−1^ of fine earth) of the soil sample on each sampling date; LT, the topsoil layer thickness (cm);BD, the bulk density of the fine earth in the topsoil (g cm^3^) estimated at the end of the experiment, the latter being computed as the ratio of the mass of fine earth (g) on the volume (cm^3^) of soil samples. We used the average value of soil density over all rotations, as this varied little (1.21 ± 0.7 g cm^−3^).

Similarly, we calculated the soil organic carbon stock (SOC_stock, expressed in t C ha^−1^) in the 0–25 cm topsoil as followed:

$$SOC\_stock=\frac{SOC\times LT\times BD}{10}$$

WithSOC, the topsoil layer C concentration (g C kg^-1^ of fine earth);

##### 4.2.2.3 Calculation of slurry amount and C content

The quantities of slurry initially expressed in m^3^ ha^−1^ were converted into t ha^-1^ on the assumption of a slurry density of 1 t m^−3^. The carbon content of the slurry was estimated at 44.1 % of the dry matter content of the slurry.

## Limitations

Most of the basic data is of good quality. The limitations concern:

1) The reduction in the number of replicates after 1991 (from 3 to 1–2 replicates depending on the rotations, with only one replicate for rotations D, E, I and J), which reduces the precision of the averages and standard deviations.
2) Observations to characterise the plant health of grasslands and maize were not extended beyond 1992. Moreover, this information is only available in French in the form of pdf files.
3) At the end of the first phase of the experiment in 1990, the permanent grassland (rotation I) was ploughed and maize was sown for one year. In addition, in 1998, when the grassland of the same rotation was affected by a severe summer drought, it was ploughed then reseeded in the autumn.
4) Soil bulk density was only measured in 2005 and was assumed to be constant over the duration of the trial in order to estimate soil organic C and N stocks.

## Ethics Statement

The authors have read the ethical requirements for publication in Data in Brief and certify that the current work does not involve human subjects, animal experiments, or any data collected from social media platforms.

## CRediT Author Statement

**Anne-Isabelle Graux**: Conceptualization, Methodology, Validation, Formal analysis, Data Curation, Writing - Original Draft, Writing - Review & Editing, Visualization, Supervision, Project administration, Funding acquisition; **Alice Cadéro**: Methodology, Validation, Data Curation, Writing - Review & Editing; **Fabien Ferchaud:** Conceptualization, Methodology, Validation, Formal analysis, Data Curation, Writing - Review & Editing, Visualization; **Françoise Vertès**: Conceptualization, Validation, Investigation, Resources, Writing - Review & Editing.

## Data Availability

Data GouvKerbernez: a long-term experiment to study the effect of different forage cropping systems on crop yields and soil organic matter in a temperate oceanic climate (Original data).
